# Supplementary material for: Effect of Material and Process Variables on Characteristics of Nitridation-Induced Self-Formed Aluminum Matrix Composites—Part 2: Effect of Nitrogen Flow Rates and Processing Temperatures
Source: Materials (Basel). 2020 Mar 8;13(5):1213. doi: 10.3390/ma13051213 (PMC7085030; doi:10.3390/ma13051213)
Supplement: Supplementary file 1 [file materials-13-01213-s001.pdf]

materials

MDPI

SUPPLEMENTARY

Effect of Material and Process Variables on Characteristics of Nitridation-Induced Self-Formed Aluminum Matrix Composites—Part 2: Effect of Nitrogen Flow Rates and Processing Temperatures

Dae-Young Kim, Pil-Ryung Cha, Ho-Seok Nam, Hyun-Joo Choi* and Kon-Bae Lee*

[1] School of Advanced Materials Engineering, Kookmin University, 02707, Seoul, South Korea; kdy6603@kookmin.ac.kr (D.-Y.K.); cprdream@kookmin.ac.kr (P.-R.C.); hsnam@kookmin.ac.kr (H.-S.N.)

* Correspondence: hyunjoo@kookmin.ac.kr (H.-J.C.); kblee@kookmin.ac.kr (K.-B.L.); Tel.: +82-2-910-4287 (H.-J.C.); +82-2-910-4230 (K.-B.L.)

Flow rate of N_2 gas (L/min)	Degree of nitridation (%)	Maximum temperature of bed (°C)	Images of manufactured composites	
			In crucible	Lathe working
1	8.7	742	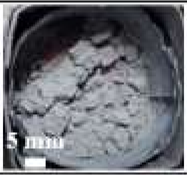	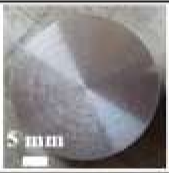
2	7.1	680	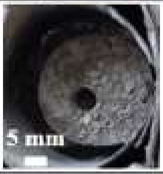	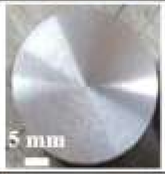
3	5.8	683	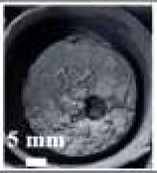	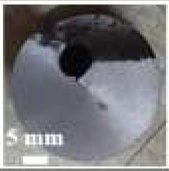
4	5.0	641	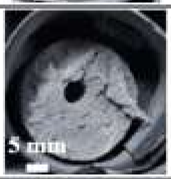	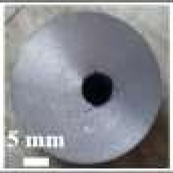

Figure S1. The degrees of nitridation, maximum powder bed temperatures, and sample images in the crucibles and after lathe working of the 20 vol% SiC (40 μm)/Al 6061 composites produced by heating at 640 °C for 1 h under various flow rates of nitrogen gas.

materials
MDPI

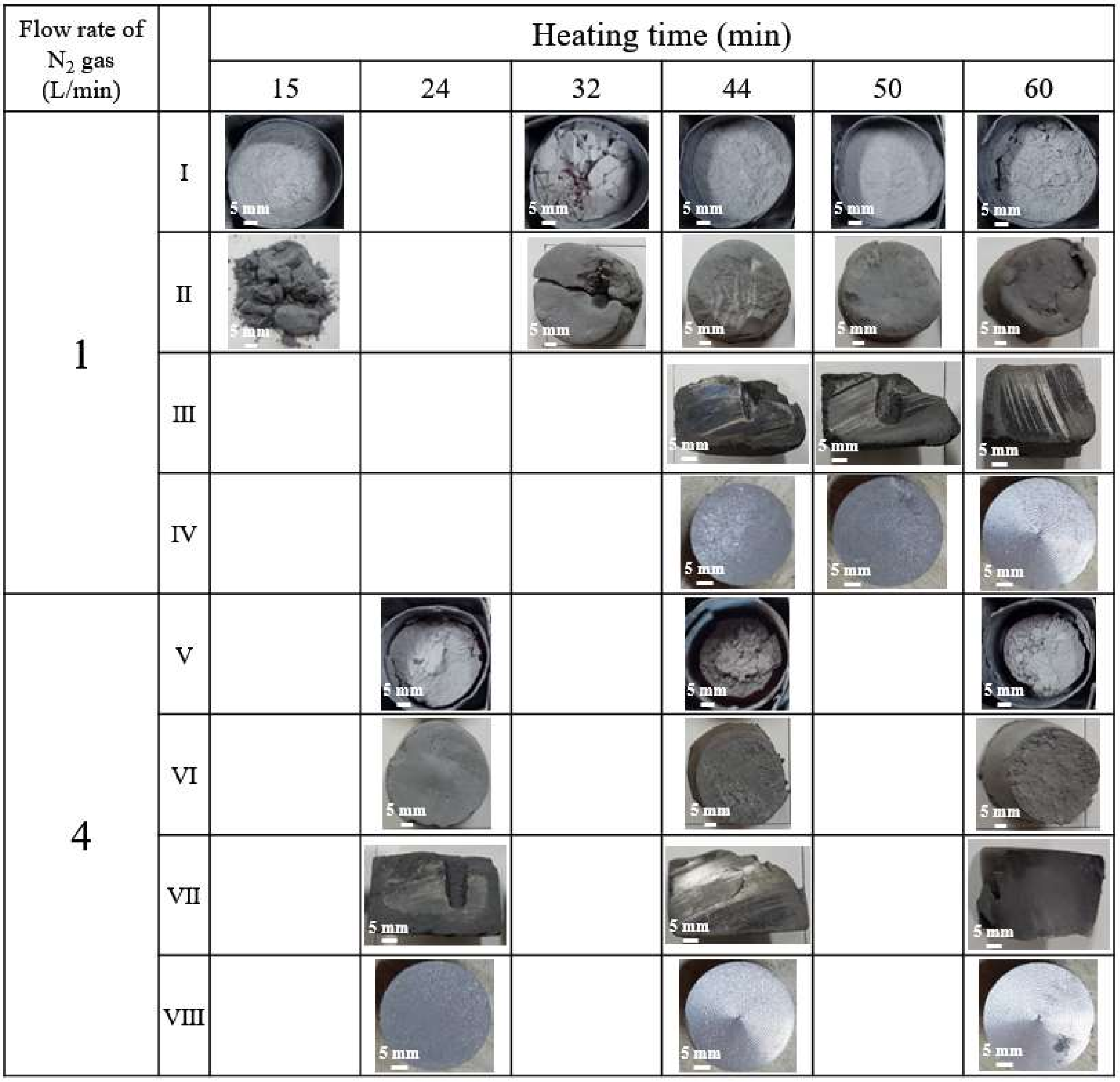

Figure S2. Photographic images of the 20 vol.% SiC (40 μm)/Al 6061 composites produced by heating with a set temperature of 640 °C for 15-60 mins under nitrogen flow rates of 1 and 4 L/min: in the crucible (rows I and V), the powder bed (rows II and VI), after cutting (rows III and VII), and after lathe working (rows IV and VIII).

materials

MDPI

	Fabrication temperature (°C)	Maximum temperature (°C)	Heating time (min)	Images of the composites	
				In crucible	Lathe working
I	610	630	90	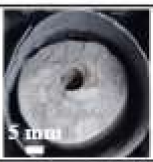	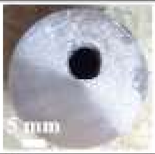
II	610	623	60	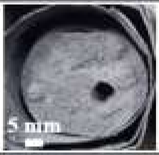	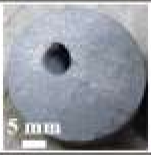
III	620	630	60	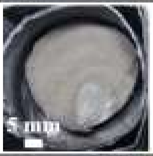	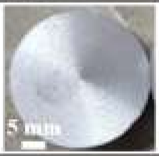
IV	630	630	60	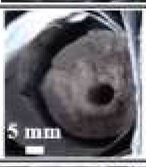	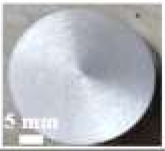
V	640	644	60	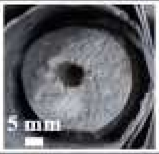	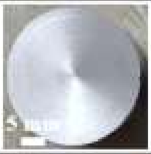
VI	650	769	60	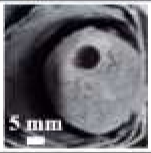	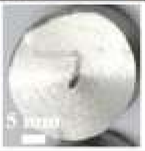

Figure S3. The maximum temperature, heating time and sample images in the crucible and after lathe working for various fabrication temperatures (610-650 °C).
